# Approximation of Nash equilibria and the network community structure detection problem

**DOI:** 10.1371/journal.pone.0174963

**Published:** 2017-05-03

**Authors:** Suciu Mihai-Alexandru, Gaskó Noémi, Lung Rodica Ioana

**Affiliations:** Centre for the Study of Complexity, Babeş-Bolyai University, Cluj Napoca, Romania; Beihang University, CHINA

## Abstract

Game theory based methods designed to solve the problem of community structure detection in complex networks have emerged in recent years as an alternative to classical and optimization based approaches. The Mixed Nash Extremal Optimization uses a generative relation for the characterization of Nash equilibria to identify the community structure of a network by converting the problem into a non-cooperative game. This paper proposes a method to enhance this algorithm by reducing the number of payoff function evaluations. Numerical experiments performed on synthetic and real-world networks show that this approach is efficient, with results better or just as good as other state-of-the-art methods.

## Introduction

Game theory plays an important role in modeling and solving real-world conflicting situations. In recent years game theory concepts have been used to address the network community structure detection problem as a game [1, 2]. This problem has multiple applications in economics, politics, sociology, biology, physics, and chemistry. Its importance arises from the fact that the community structure offers structural and functional information about the network that cannot be derived from other indicators. Intuitively, a community is described as a group of nodes that are highly connected to each other and sparsely connected to the outside. One of the main challenges related to this problem comes from the lack of a universally accepted formal definition for the community structure encompassing all aspects that emerge from the intuitive description above. A comprehensive survey of the problem, including possible definitions for the community structure, can be found in [3].

Game theory models strategic and conflicting situations offering a variety of solution concepts that are known as *game equilibria*. The most popular example is the Nash equilibrium, which models a situation of a game in which no player has any incentive for unilateral deviation [4]. The Nash equilibrium has been used to characterize the network community structure by considering a game in which nodes are players that strategically choose their community to maximize their payoffs. But this approach leads to multi-player games that are difficult to solve when the payoff functions do not have nice mathematical properties. A heuristic model that computes Nash equilibria for such games has been proposed and adapted for the community detection problem in [1, 5] for static unweighted networks, and in [2] for dynamic networks. The model is based on extremal optimization and a generative relation for strategy profiles that enables their comparison [6], guiding the search towards game equilibria.

This paper proposes a reduced version of the generative relation—requiring less payoff function evaluations—for Nash equilibria characterization. We first provide a formal proof that this relation, which only takes into account a fraction of players when comparing two strategy profiles, can also characterize the Nash equilibria of the game. Numerical experiments are performed to evaluate this approach; results are compared with those provided by other state-of-art methods from the literature.

## Methods

The community structure detection problem is usually described as searching for a partition over the set of network nodes such that nodes within each set are *more* connected to each other than to nodes in other sets of the partition. While the concept of community as a social term is straight forward, its formalization is not trivial. Several attempts to define it can be found in the literature [7–14], but none of them captures all aspects embedded in the intuitive definition above.

A popular attempt defines the community structure as the optimum value of a fitness function defined to capture the network structure; the advantage of considering such a function is that heuristic methods can further be used to compute the optimal structure. Examples of such functions are: the *modularity* [15], the *community score* [16], the *community fitness* [17], and the *modularity density* [18]. However, none of these functions represent the real structure for all types of networks as their optimal values do not always correspond to the known community structure [19, 20].

A recent approach considers the community structure as the equilibrium of a mathematical game. Game theoretic approaches usually transform the community detection problem into a game in which nodes have the roles of players that have to choose a community to maximize their payoffs. The first game-based models use the modularity function [15] to derive payoffs for players [21, 22]. Game theoretic models have been proposed mostly for networks with overlapping communities [21, 23, 24] and for dynamic networks [2, 25]; models based on cooperative games theory have been proposed in [26–28]; other game based approaches can be found in [1, 5, 29].

In [29] the game is defined also over the set of nodes, i.e. nodes are players that maximize a payoff computed as the number of neighbors the node has in a community and a fraction of its neighbors that are connected among themselves. The authors propose a method to compute the Nash equilibria of this game, called Nash Stability based Community Detection (NashCoDe). Numerical results are reported in terms of modularity and coverage for a set of real-world networks.

Another formal game theoretic approach for unweighted networks constructs a hedonic game [22] in which nodes are the players and payoffs are computed using a local modularity function. The advantage of this approach is that the properties of the payoffs guarantee the existence of a Nash equilibrium for this game. The authors also design a community detection algorithm (CDG) that alternates three mechanisms to find the equilibrium. Numerical results are reported on a set of real-world networks and synthetic networks.

### Reduced nash ascendancy

Consider a game Γ = (*N*, *S*, *U*), where *N* is the set of players, *N* = {1, …, *n*}, $S=\prod_{i=1}^{n}S_{i}$ is the set of strategy profiles of the game with *S*_*i*_ the set of strategies of player *i*, and $U={\left\{u_{i}\right\}}_{i\in N},u_{i}:S\to R$ the player’s payoff functions. A Nash equilibrium (NE) is a strategy profile such that there is no player that can improve his payoff by changing his strategy while all other players maintain theirs unchanged. Currently there are a plethora of methods that compute the Nash equilibria of a game. Among them an interesting class is formed of the methods based on evolutionary computation as they are supposed to be highly adaptable to various payoff functions and, more important, do not require nice mathematical properties for them [6, 30–32]. In [6] the basis for the direct search for Nash equilibria by evolutionary algorithms is designed by the proposal of a generative relation called the Nash ascendancy relation that can be used to characterize and furthermore compare strategy profiles of the game with respect to NEs. A quality operator k:S×S→N computed as the number of players that would improve their payoffs by unilaterally switching their strategies from one strategy profile to the other is used:

$$\begin{array}{c} k\left(s,q\right)=card\{i\in N|u_{i}\left(s\right)\leq u_{i}\left(q_{i},s_{-i}\right),s_{i}\neq q_{i}\}, \end{array}$$ (1)

where *card*{} denotes the cardinality of a set, and

$$\begin{array}{c} \left(q_{i},s_{-i}\right)=\left(s_{1},s_{2},\ldots ,s_{i-1},q_{i},s_{i+1},\ldots ,s_{n}\right). \end{array}$$

Operator *k* is used to define the Nash generative relation that enables the comparison of two strategy profiles with respect to the NE:

- if *k*(*s*, *q*) < *k*(*q*, *s*) we say that *s* Nash *ascends* (or Nash dominates) *q*, and that *s* is considered *better* than *q* in Nash sense;
- if *k*(*s*, *q*) > *k*(*q*, *s*) we say that *s* is *ascended* (or Nash dominated) by *q* in Nash sense;
- if *k*(*s*, *q*) = *k*(*q*, *s*) then *s* and *q* are indifferent to each other in Nash sense.

A strategy profile that is not ascended/dominated by any other is called in this context Nash non-ascended/non-dominated (NND). In [6] it is shown that if a game has at least one NE, then the NND set coincides with the set of NEs of the game. In the absence of Nash equilibria, the NND set can offer useful information about practical solutions that have properties similar to Nash equilibria to problems that were not previously approached by game theoretic tools.

However, the ascendancy relation requires up to 2 ⋅ *n* payoff function evaluations for each pair (*s*, *q*); this can become computationally expensive when dealing with large games. In this paper we are exploring the possibility of replacing the Nash ascendancy relation described above with a reduced version [33], which requires less payoff function evaluations when comparing two strategy profiles. The main result of this section is the formal proof that the reduced version also characterizes the Nash equilibria of the game, i.e. in the presence of the Nash equilibrium the set of non-dominated solutions with respect to this relation is equal to the set of equilibria.

The reduced relation considers only a fraction *p* of the nodes when computing *k*, where *p* ∈ (0, 1] such that *n*_*p*_ = [*p* ⋅ *n*] > 0, where [⋅] denotes the integer part. We denote by *I*_*p*_ ⊂ *N*, *I*_*p*_ = {*i*_1_, *i*_2_, …, *i*_*n*_*p*__} any set of *n*_*p*_ players, and $I_{p}\subset P\left(N\right)$ the set of all subsets *I*_*p*_ of *N* having size *n*_*p*_. Operator $k_{p}:S\times S\times I_{p}\to R$ is defined as:

$$\begin{array}{c} k_{p}\left(s,q,I_{p}\right)=card\left\{i\in I_{p}|u_{i}\left(s\right)\leq u_{i}\left(q_{i},s_{-i}\right),s_{i}\neq q_{i}\right\}. \end{array}$$ (2)

**Definition 1**. *We say that strategy profile* *s* *p*–***Nash ascends*** *q* *with respect to* $I_{p}\in I_{p}$ *if*

$$\begin{array}{c} k_{p}\left(s,q,I_{p}\right)<k_{p}\left(q,s,I_{p}\right). \end{array}$$ (3)

If *p* = 1, the *p*–Nash ascendancy relation is the same as the Nash ascendancy relation.

**Definition 2**. *Strategy* *s* ∈ *S* *is* *p*–*Nash non-ascended* (*p*–*Nash non-dominated*) *if there is no* *q* ∈ *S* *and no* $I_{p}\in I_{p}$ *such that*

$$\begin{array}{c} k_{p}\left(q,s,I_{p}\right)<k_{p}\left(s,q,I_{p}\right). \end{array}$$

We denote the set of *p*–Nash non-dominated solutions of Γ as *p*NND.

**Proposition 1**. *For any* *s*, *q* ∈ *S* *we have*:

$$\begin{array}{c} \sum_{I_{p}\in I_{p}}k_{p}\left(s,q,I_{p}\right)=C_{n-1}^{n_{p}-1}k\left(s,q\right), \end{array}$$ (4)

*where* $C_{n-1}^{n_{p}-1}$ *denotes the binomial coefficient*.

*proof*. Let *k*(*s*, *q*) = *l* and *I* = {*i*_1_, …, *i*_*l*_} the set of *l* players that can improve their payoffs by unilaterally deviating from *s* to *q*. Then each player *i*_*j*_ belongs to $C_{n-1}^{n_{p}-1}$ sets $I_{p}\in I_{p}$ and we can write:

$$\begin{array}{c} \sum_{I_{p}\in I_{p}}k_{p}\left(s,q,I_{p}\right)=C_{n-1}^{n_{p}-1}\cdot l=C_{n-1}^{n_{p}-1}k\left(s,q\right). \end{array}$$

**Proposition 2**. *Any* *p*–*Nash non-dominated solution is a Nash non-dominated solution*.

*proof*. Let *s* ∈ *p*NND, and *q* ∈ *S*. Then, based on Prop. 1, we have

$$\begin{array}{c} k\left(s,q\right)=\frac{1}{C_{n-1}^{n_{p}-1}}\sum_{I_{p}\in I_{p}}k_{p}\left(s,q,I_{p}\right)\leq \frac{1}{C_{n-1}^{n_{p}-1}}\sum_{I_{p}\subset N}k_{p}\left(q,s,I_{p}\right)=k\left(q,s\right) \end{array}$$ (5)

meaning that *s* ∈ *NND*.

This result can be extended in the following manner:

**Proposition 3**. *Let* *p*, *r* ∈ [0, 1] *with* *r* > *p*. *Then* $\forall I_{r}\in I_{r}$:

$$\begin{array}{c} k_{r}\left(s,q,I_{r}\right)=\frac{1}{C_{n_{r}-1}^{n_{p}-1}}\sum_{I_{p}\subset I_{r}}k_{p}\left(s,q,I_{p}\right) \end{array}$$ (6)

*proof*. Let *k*_*r*_(*s*, *q*, *I*_*r*_) = *l*, *l* < *n*_*r*_ and *I* = {*i*_1_, …, *i*_*l*_} the set of *l* players from *I*_*r*_ that can improve their payoffs by unilaterally deviating from *s* to *q*. Then each player *i*_*j*_ belongs to $C_{n_{r}-1}^{n_{p}-1}$ subsets *I*_*p*_ ⊂ *I*_*r*_ and we can write:

$$\begin{array}{c} \sum_{I_{p}\subset I_{r}}k_{p}\left(s,q,I_{p}\right)=C_{n_{r}-1}^{n_{p}-1}\cdot l=C_{n_{r}-1}^{n_{p}-1}k_{r}\left(s,q,I_{r}\right). \end{array}$$

By considering *r* = 1 in Propositionn 3 we obtain the result in Proposition 1. Moreover, based on proposition 3 we can also formulate the following result:

**Proposition 4**. *Let* *p*, *r* ∈ [0, 1] *with* *r* > *p*. *Then any* *p*–*Nash non-dominated solution is also a* *r*–*Nash non-dominated solution*.

*proof*. The proof is straight-forward from Proposition 3 and similar to that of Proposition 2.

**Proposition 5**. *Any Nash equilibrium of* Γ *is a* *p*-*Nash non-ascended solution*, *p* ∈ (0, 1].

*proof*. The proof is direct from the definition of the NE.

From Propositions 2, 5, and based on [6] it follows that *p*–Nash non-dominated solutions are also Nash equilibria for the game, if such an equilibrium exists. Thus, approximating *p*–Nash non-dominated solutions leads to the same results as approximating Nash non-dominated solutions by using the Nash ascendancy relation.

### Community structure detection game

In this paper we approach the game proposed in [5] with the reduced variant of the generative relation used to characterize Nash equilibria. Given a network *G* = (*V*, *E*), where *V* is the set of vertices and *E* is the set of edges, consider the following game $\tilde{\Gamma }=\left(N,S,U\right)$ [5], where:

- *N* is the set of players represented by network nodes, *N* = *V* = {1, 2, …, *n*}; where *n* is the network size;
- *S* = *S*_1_ × *S*_2_ × ⋯ × *S*_*n*_ the set of strategy profiles, and *S*_*i*_ the set of strategies of player *i*; in this case *S*_*i*_ represents the set of possible communities node *i* may choose from; an element *s* ∈ *S* is a possible community structure; we write *s* = (*C*_1_, *C*_2_, …, *C*_*n*_), *C*_*i*_ represents the community of node *i*, *i* ∈ {1, 2, …, *n*}; we denote strategies *C*_*i*_ by using integers, *C*_*i*_ ∈ {1, …, *C*_*max*_} where *C*_*max*_ is a maximum value expected for the number of communities in the network, and with $C_{w}=\left\{i\in N|C_{i}=w\right\}$, *w* = 1, …, *C*_*max*_ the community containing all nodes having *C*_*i*_ = *w*, *i* ∈ {1, …, *n*}; for example, *C*_*i*_ = 2 indicates that player *i* belongs to community 2 with all other nodes *j* ∈ *N* with *C*_*j*_ = 2, and we will denote by $C_{2}$ the community containing all nodes *i* ∈ *N* with *C*_*i*_ = 2.
- *U* is the payoff function, *U* = (*u*_1_, *u*_2_, …, *u*_*n*_) where $u_{i}:S\to R$ is the payoff function of player *i* ∈ *N*, computed as the node’s contribution to its community $C_{w}$, where *C*_*i*_ = *w* [1, 17]. We compute the payoff *u*_*i*_(*C*_1_, *C*_2_, …, *C*_*i*_, …, *C*_*n*_) of node *i* relative to community $C_{w}$ as the difference between the community fitness of $C_{w}$ with node *i* included in it and its fitness when *i* is excluded:
  $$\begin{array}{c} u_{i}\left(C_{1},C_{2},\ldots ,C_{i},\ldots ,C_{n}\right)=f\left(C_{w}\cup \left\{i\right\}\right)-f\left(C_{w}\setminus \left\{i\right\}\right),\;\text{with}\;C_{i}=w. \end{array}$$ (7)
  The fitness f(C) of community C in Eq (7) [17] is:
  $$\begin{array}{c} f\left(C\right)=\frac{\sum_{j\in C}k_{j}^{in}}{{\left(\sum_{j\in C}\left(k_{j}^{in}+k_{j}^{out}\right)\right)}^{\alpha }}, \end{array}$$
  where $k_{j}^{in}$ is the internal degree of node *j* in community C (the number of links connecting node *j* to other nodes in C), $k_{j}^{out}$ is the external degree of node *j* with respect to community C (the number of links connecting node *j* to nodes outside of C), and *α* is a parameter controlling the size of the community. In what follows we will consider *α* = 1.

**Fig 1:**
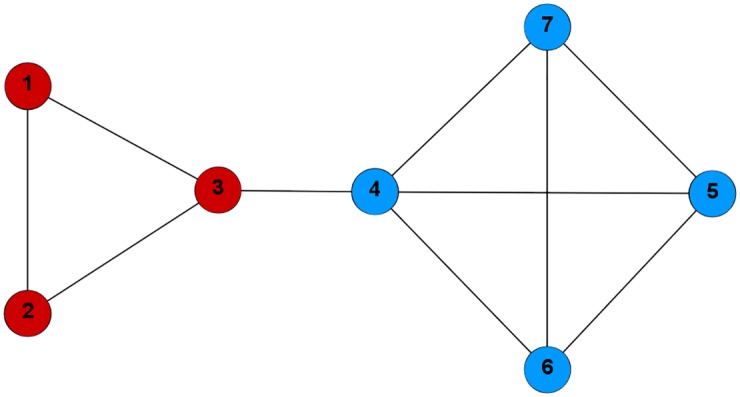
The communities $C_{1}=\left\{1,2,3\right\}$ (red), $C_{2}=\left\{4,5,6,7\right\}$ (blue) form the strategy profile *s* = (1, 1, 1, 2, 2, 2, 2).

**Example 1**. *Consider the network in* Fig 1 *and two different community structures* (Figs 1 *and* 2).

**Fig 2:**
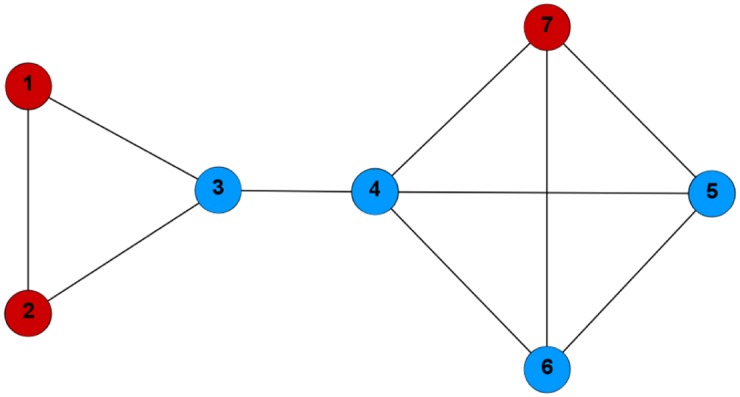
The communities $C_{1}^{\prime }=\left\{1,2,7\right\}$ (red), $C_{2}^{\prime }=\left\{3,4,5,6\right\}$ (blue) form the strategy profile *q* = (1, 1, 2, 2, 2, 2, 1).

*In the first situation* (Fig 1) *we have*:

$$\begin{array}{c} f\left(C_{1}\right)=\frac{2+2+2}{\left(2+0)+(2+0)+(2+1\right)}=\frac{6}{7},\;f\left(C_{2}\right)=\frac{12}{13} \end{array}$$

*and the payoff of the third node (player) is the following*:

$$\begin{array}{c} u_{3}\left(1,1,1,2,2,2,2\right)=\frac{6}{7}-\frac{1+1}{\left(1+1)+(1+1\right)}=\frac{5}{14}. \end{array}$$

*In the second case* (Fig 2):

$$\begin{array}{c} f\left(C_{1}^{\prime }\right)=\frac{2}{7},\;f\left(C_{2}^{\prime }\right)=\frac{8}{13}. \end{array}$$

*The payoff for the third player is*:

$$\begin{array}{c} u_{3}\left(1,1,2,2,2,2,1\right)=\frac{8}{13}-\frac{2+2+2}{\left(2+2)+(2+1)+(2+1\right)}=\frac{1}{65}. \end{array}$$

*We have* *k*(*s*, *q*) = 0, *because* *u*_3_(*s*) ≰ *u*_3_(1, 1, 2, 2, 2, 2, 2) *and* *u*_7_(*s*) ≰ *u*_7_(1, 1, 1, 2, 2, 2, 1) (*we need to verify only these two inequalities, because* *s*_*i*_ = *q*_*i*_ *for* *i* = 1, 2, 4, 5, 6). *On the other hand* *k*(*q*, *s*) = 2, *because* *u*_3_(*q*) ≤ *u*_3_(1, 1, 1, 2, 2, 2, 1) *and* *u*_7_(*q*) ≤ *u*_7_(1, 1, 2, 2, 2, 2, 2). *So we can say that* *s* *Nash ascends* *q*, *or that* *s* *is better than* *q* *in Nash sense*.

*If we consider* *p* = 0.50 *and* *I*_*p*_ = {3, 1, 5} *we obtain* *k*_*p*_(*s*, *q*, *I*_*p*_) = 0 *and* *k*_*p*_(*q*, *s*, *I*_*p*_) = 1, *which means that* *s* 0.50-*Nash ascends strategy profile* *q* *with respect to* *I*_*p*_.

While we cannot guarantee the existence of the Nash equilibrium for this game [21, 23], we can attempt to approximate it by using a search heuristic with the generative relation for characterization of Nash equilibria [6]. Particularly, the assumption that the NNDs of game $\tilde{\Gamma }$ represent the community structure of the network was empirically tested in [1] by means of numerical experiments performed on synthetic and real-world networks by using the Mixed Nash Extremal Optimization (MNEO) algorithm. MNEO showed superior performance compared to other methods in identifying the community structure for some networks considered difficult.

### Computation method: *p*-Mixed Nash Extremal Optimization

In order to test the *p*-ascendancy relation, we adapt the Mixed Nash Extremal Optimization [1] to use of the *p*–ascendancy relation instead of the Nash ascendancy. Thus, the only modification made to MNEO, which is described in what follows, is the replacement, in line 4 of Algorithm 1, of the Nash ascendancy relation with the *p*–Nash ascendancy relation (Definition 1) with respect to a randomly generated set *I*_*p*_. The particular set $I_{p}\in I_{p}$ of nodes that are tested whenever two individuals are compared by using the reduced Nash ascendancy relation is randomly generated each time a comparison is performed. *p* is a parameter of the method and we denote by *p*MNEO the reduced version of MNEO.

#### Encoding

Each individual *P*_*j*_ encodes a network structure as a vector of integers of size *n*, *P*_*j*_ = (*p*_*j*_1__, …, *p*_*j*_*n*__), for node *i*, *p*_*j*_*i*__ represents the community of node *i* for individual *j*. Each individual searches for a fixed number of communities, which is set within a minimum possible value, *C*_*min*_, and a maximum one, *C*_*max*_.

#### Populations

*p*MNEO evolves a population *P* and an archive *A*; individuals in *A* preserve the best solutions found so far by each individual in *P*. Paired individuals (*P*_*j*_, *A*_*j*_) from the two populations follow the rules of extremal optimization: individual *P*_*j*_ explores the search space; the best value found by *P*_*j*_ is preserved in *A*_*j*_, $j=\bar{1,M}$, where *M* is the size of the two populations.

#### Extremal optimization

Individuals in population *P* explore the search space following the rules of an EO-based iteration: for each individual *P*_*j*_, the *ℓ* nodes having the worst fitnesses are randomly assigned to different communities. If the newly obtained partition $P_{j}^{\prime }$ *p*-Nash ascends the corresponding archive member *A*_*j*_ relative to a randomly generated *I*_*p*_ ∈ *N*, it will replace it. If not, the search continues next iteration from $P_{j}^{\prime }$ (Algorithm 1).

**Algorithm 1 The Extremal Optimization iteration.**

**Input:** Current population *P*, archive *A*;

**Parameters:** number of nodes to be changed *ℓ*;

Archive member *A*_*j*_, $j=\bar{1,M}$, preserves the best solution found so far by *P*_*j*_.

1: **for** each individual *P*_*j*_ in *P* **do**

2:  Select the *ℓ* nodes having the lowest payoffs from *P*_*j*_;

3:  Randomly assign another community to each selected node—create offspring $P_{j}^{\prime }$;

4:  **if** $P_{j}^{\prime }$ *p*–Nash ascends *A*_*j*_ (Alg. 2) **then**

5:   set $A_{j}:=P_{j}^{\prime }$;

6:  **end if**

7:  set $P_{j}=P_{j}^{\prime }$

8: **end for**

The *p*-Nash ascendancy relation is implemented as described in Algorithm 2. An important aspect of this relation is that at line 4 it tests if node *i* is in the same community in both *P* and *A*: if it is so, we do not attempt to compare payoff values, even though they might be different because of the rest of the structures, i.e. equal strategies in *P* and in *A* do not imply equal payoffs, but they are not compared as they are not included in *k*_*p*_(*P*, *A*, ⋅) Eq (3).

**Algorithm 2** *p*-**Nash ascendancy relation**

**Input:** Individuals *P* and *A*; Probability *p*

**Output:** 1 if *P* *p*-Nash ascends *A*, -1 if *A* Nash ascends *P*, 0 if they are indifferent to each other.

1: Set *k*_*p*_(*P*, *A*) = 0;*k*_*p*_(*A*, *P*) = 0;

2: **for** *h* = 0;*h* < *n*_*p*_ = *np*;*h*++ **do**

3:  Randomly select node *i*;

4:  **if** *P*_*i*_ < >*A*_*i*_ **then**

5:   **if** *u*_*i*_(*P*)<*u*_*i*_(*A*_*i*_, *P*_−*i*_) **then**

6:    *k*_*p*_(*P*, *A*)++;

7:   **end if**

8:   **if** *u*_*i*_(*A*)<*u*_*i*_(*P*_*i*_, *A*_−*i*_) **then**

9:    *k*_*p*_(*A*, *P*)++;

10:   **end if**

11:  **end if**

12: **end for**

13: **if** *k*_*p*_(*P*, *A*)<*k*_*p*_(*A*, *P*) **then**

14:  return 1; (*P* *p*–Nash ascends *A*)

15: **else**

16:  **if** *k*_*p*_(*A*, *P*)<*k*_*p*_(*P*, *A*) **then**

17:   return -1; (*A* *p*–Nash ascends *P*)

18:  **else**

19:   return 0; (*P* and *A* are indifferent to each other)

20:  **end if**

21: **end if**

#### Diversity preserving mechanism

*p*MNEO uses the mixing mechanism used by MNEO designed to preserve the node degrees in the network and proposed in [34]. Two links are randomly chosen, deleted, and the nodes are re-connected in a different manner. Links are selected randomly with a probability *ρ* controlling the magnitude of change in the network. *p*MNEO alternates the search on the original network for Λ iterations with the search on the modified network for *λ* iterations.

#### Outline of *p*MNEO

Algorithm 3 presents an outline of *p*MNEO.

**Algorithm 3** Outline of *p*- **Mixed Network Extremal Optimization.**

1: Randomly initialize all individuals in *P* and *A*;

2: Evaluate all individuals in *P* and *A*;

3: **for** *NrGen* from 0 to *MaxGen* **do**

4:  Set $\ell_{ngen}=\max\left\{1,\left[\frac{1}{10}\cdot n\cdot {\left(n-2\right)}^{-\frac{NrGen}{MaxGen}}\right]\right\}$

5:  Run an EO iteration (alg. 1);

6:  **if** Λ iterations were performed on the original network **then**

7:   Mix network with probability *ρ*;

8:   Randomly re-initialize population *A*;

9:   Evaluate all individuals in *P* and *A*;

10:  **end if**

11:  **if** Network is mixed and *λ* iterations were performed **then**

12:   Restore network to original structure;

13:   Randomly re-initialize population *A*;

14:   Evaluate all individuals in *P* and *A*;

15:  **end if**

16: **end for**

17: **Output:** individual from *A* having the best modularity;

#### Output

The individual with best modularity *Q* [15]. The modularity function is not used to guide the search, but only to select the best individual in the final population and is computed as:

$$\begin{array}{c} Q=\frac{1}{2m}\sum_{ij}\left(A_{ij}-\frac{d_{i}d_{j}}{2m}\right)\delta \left(C_{i},C_{j}\right), \end{array}$$ (8)

where the sum runs over all pairs of vertices *i* and *j*, *A* is the adjacency matrix, *m* the total number of links in the network, *d*_*i*_ the degree of node *i*, *C*_*i*_ the community of node *i* and *δ*(*C*_*i*_, *C*_*j*_) equals 1 if nodes *i* and *j* belong to the same community and 0 otherwise. When two community structures are compared, a higher modularity value presumably indicates a better solution.

#### Parameters

*p*MNEO uses the following parameters:

- fraction *p* of nodes that are evaluated during the *p*–Nash ascendancy relation test;
- Population size *M*;
- Maximum number of iterations *MaxGen*;
- *ρ*—network mixing probability;
- Λ and *λ*—number of iterations MNEO performs the search on the original network and the modified one, respectively;
- *C*_*min*_ and *C*_*max*_—minimum and maximum number of communities searched for.

## Results

We illustrate the effect of using the reduced generative relation within pMNEO by means of numerical experiments performed on a Cournot oligopoly and on synthetic and real-world networks with known community structures.

### Experimental set-up

#### Cournot oligopoly

The Cournot oligopoly is a well known competitive market game, suitable as a benchmark for testing equilibria detection methods as it presents one known equilibrium, and it is scalable.

Let *q*_*i*_, *i* = 1, …, *n* quantities of an homogeneous product—produced by *n* companies respectively. We consider the market clearing price as

$$\begin{array}{c} P(Q)=a-Q, \end{array}$$

where *Q* is the aggregate quantity on the market. We have

$$\begin{array}{c} P\left(Q\right)=\left\{ \begin{array}{cc} a-Q, & \text{for}\;Q<a,\\ 0, & \text{for}\;Q\geq a. \end{array}\right. \end{array}$$

If the total cost for the company *i* of producing quantity *q*_*i*_ is *C*(*q*_*i*_) = *cq*_*i*_, i.e. there are no fixed costs and the marginal cost *c* is constant, *c* < *a*, and that companies choose their quantities simultaneously, the payoff for the company *i* is its profit:

$$\begin{array}{c} u_{i}\left(q_{1},q_{2},...,q_{n}\right)=q_{i}P\left(Q\right)-C\left(q_{i}\right). \end{array}$$

If $Q=\sum_{i=1}^{n}q_{i},$ the Cournot oligopoly has one Nash equilibria that can be computed by

$$\begin{array}{c} q_{i}=\frac{a-c}{n+1},\forall i\in \left\{1,...,n\right\}. \end{array}$$

We will use the Cournot oligopoly to illustrate the behavior of pMNEO when considering different probabilities for the generative relation. However, since convergence to local equilibria is not an issue in this case, we do not need the mixing mechanism to ensure diversity. We denote by pMNEO^−^ the algorithm used to perform numerical experiments on the Cournot oligopoly.

**Parameter settings for the Cournot oligopoly** We tested pMNEO^−^ for 10, 50, 100, 500, and 1000 players, by using 10 individuals encoding a strategy profile of the game; in our experiments we set *a* = 24, *c* = 9, *MaxGen* = 10^4^, *S* = [0, 10]^*n*^. Tested values for probability *p* are 0.25, 0.50, 0.75, and 1. Each generation we compute and report the distance to the Nash equilibrium of the game.

#### Community structure detection

**Benchmarks** *p*MNEO is tested on the GN and LFR synthetic benchmarks [35, 36] (generated using the code available at https://sites.google.com/site/andrealancichinetti/software) and on four real world datasets with known community structure: the bottle-nose *dolphin* network [37], the *football* network [36], the Zachary *karate* club network [38], and the *books* about US politics network (http://www.orgnet.com, last accessed 9/3/2015). A set of three networks with unknown community structure is also tested: *risk map* [39], *jazz* [40], and *contiguous USA* [41].

The GN benchmark consists of eight network sets having 128 nodes grouped in 4 communities of 32 nodes; each node has a degree of 16. Each set is characterized by the number of links a node has outside its community, *z*_*out*_ ∈ {1, 2, …, 8}, and contains 30 networks.

Three sets of LFR benchmarks were generated: one set having 128 nodes, and 2 with 1000 nodes, each of them consisting of 5 sets containing 30 networks with different mixing parameters *μ* ∈ {0.1, 0.2, …, 0.5}. The mixing parameter *μ* is the ratio between the number of links a node has outside its community and its degree. The LFR parameters are: average vertex degree 20, maximum vertex degree 50, community size [10, 50] for the S (*small*) set with 128 and 1000 nodes, and [20, 100] for the B (*big*) set with 1000 nodes.

**Parameters** *p*MNEO has only one specific parameter apart from MNEO, the fraction *p* of nodes used within the *p*-Nash ascendancy relation. Four values of *p* were tested: 25%, 50%, 75%, and 100%. When *p* = 100%, *p*MNEO is the same with MNEO. The termination condition was set as the maximum number of generations for each benchmark, indicated with the numerical results for each set. In order to assess if there are differences between results obtained by using these values, all other parameters are maintained constant (with values indicated in [1]), i.e.: population size 50, probability *ρ* for the network mixing mechanism 0.02, Λ = 30, and *λ* = 10. The parameters *C*_*min*_ and *C*_*max*_ were set to include the real number of communities and to allow 25% of the population to use it as a parameter.

**Comparison with other methods** The results obtained by *p*MNEO are compared to the following state-of-the-art algorithms: OSLOM [42], Infomap [43], Modularity optimization (ModOpt) [44], and the Louvain method [45] (for these algorithms we use the code and parameter settings available at https://sites.google.com/site/andrealancichinetti/software, downloaded on March 2014). Each of these algorithms are known to be fast and efficient.

**Performance measure** A reliable performance measure for assessing the quality of the results offered by different community detection methods is the normalized mutual information (NMI)^1^ [17]. The NMI can be used when the community structure is available; the higher the value of the NMI, the better the solution; the maximum value of 1 indicates that the compared structures are identical. Thus, if a result is compared to the known structure, a NMI value of 1 indicates that the correct structure has been found.

NMI values obtained by different methods are compared by using the Wilcoxon signed-rank non-parametric test. Thus we consider the NMIs reported in 30 independent runs for each real network and for the 30 networks of each GN or LFR set, by each method. The Wilcoxon sign rank assesses if there is a significant difference between two sample means; the null hypothesis that two samples come from the same population can be rejected with a level of significance *α* = 0.05 if the computed *p*-value is smaller than 0.05.

Also in order to evaluate the quality of the reported solution from the equilibria point of view, we verify for each one if there are any players that would improve their payoffs by unilateral deviation, and report the fraction of this number to the number of nodes in the network. In this manner we can assess if *p*MNEO actually computes Nash equilibria of the community structure detection game.

### Results and discussion

#### Cournot oligopoly

**Fig 3:**
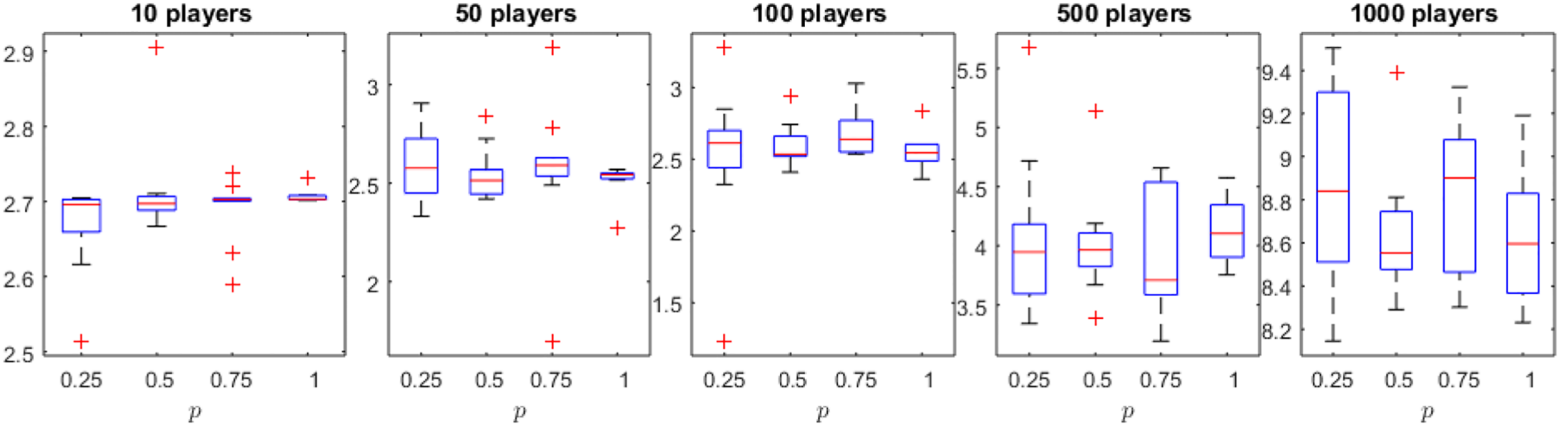
Distance to Nash equilibrium for the Cournot oligopoly and different *p* values. The null hypothesis that there is no statistical difference between means of results could not be rejected by using a Wilcoxon sum-rank test (0.05 significance level).

**Fig 4:**
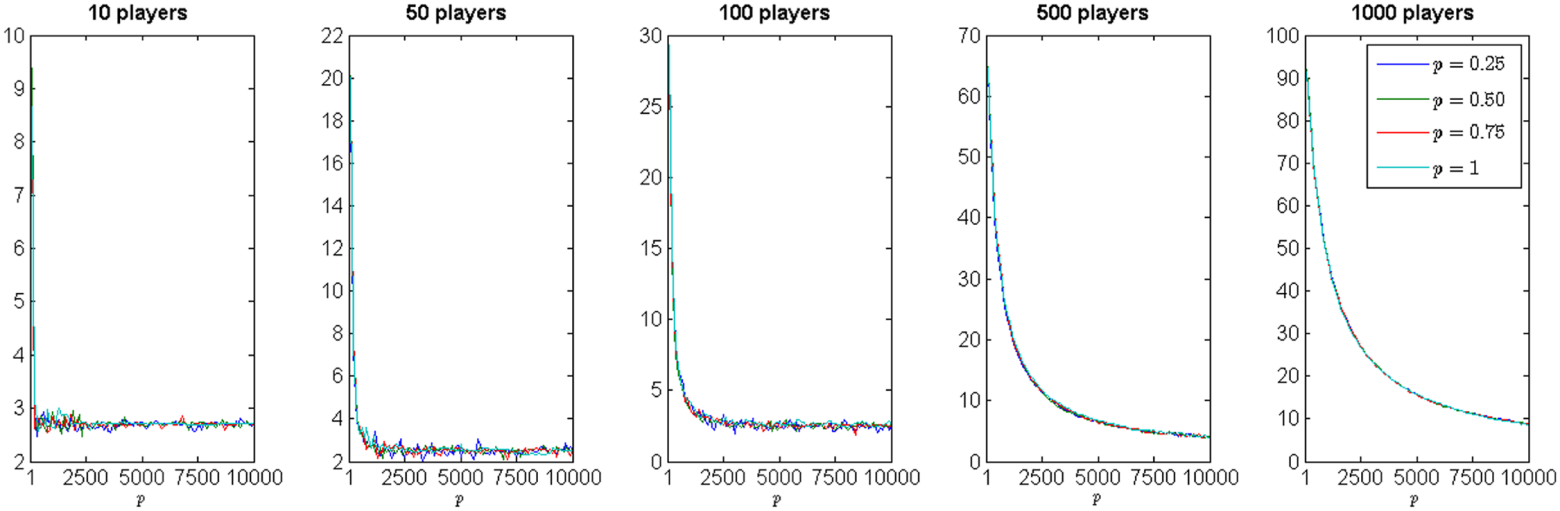
Evolution of the distance to NE in all cases: again no significant diference between the four *p* values is observed.

**Fig 5:**
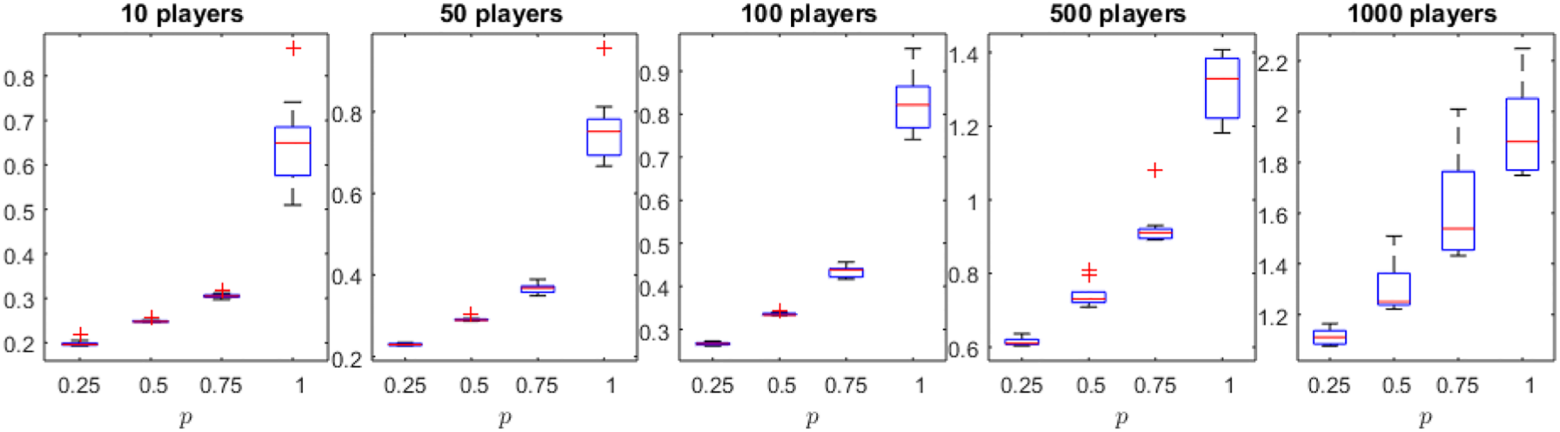
Duration of the runs (in sec.). The null hypothesis that differences between mean values are not significant was rejected by using a Wilcoxon sum-rank test with a significance level of 0.05.

The results reported by *p*MNEO^−^ on the Cournot oligopoly are presented in Figs 3–5. Fig 3 presents boxplots of minimum distance to NE for all the player settings. There are no significant differences between results reported when using different *p* values. Fig 4 complements this information by showing the evolution of the distance to NE with similar behavior for all *p* values. Fig 5 however, shows significant differences between the duration of runs (in seconds) for all *p* values, supporting the hypothesis that the use of the reduced generative relation yields faster, but just as good, results for the Cournot oligopoly.

#### Community structure detection

NMI values computed for the results obtained by *p*MNEO by using the four different values of *p* as well as with the other methods for all considered networks are presented as boxplots accompanied by black-white matrices representing Wilcoxon *h*-values for each pair of methods tested in order to make the assessment about the statistical significance of possible differences straightforward (a white square in the matrix indicate no statistical differences in results while a black one indicates significant differences). The numbers 1 to 8 indicate the methods as they are ordered in the box-plots in the left.

**Fig 6:**
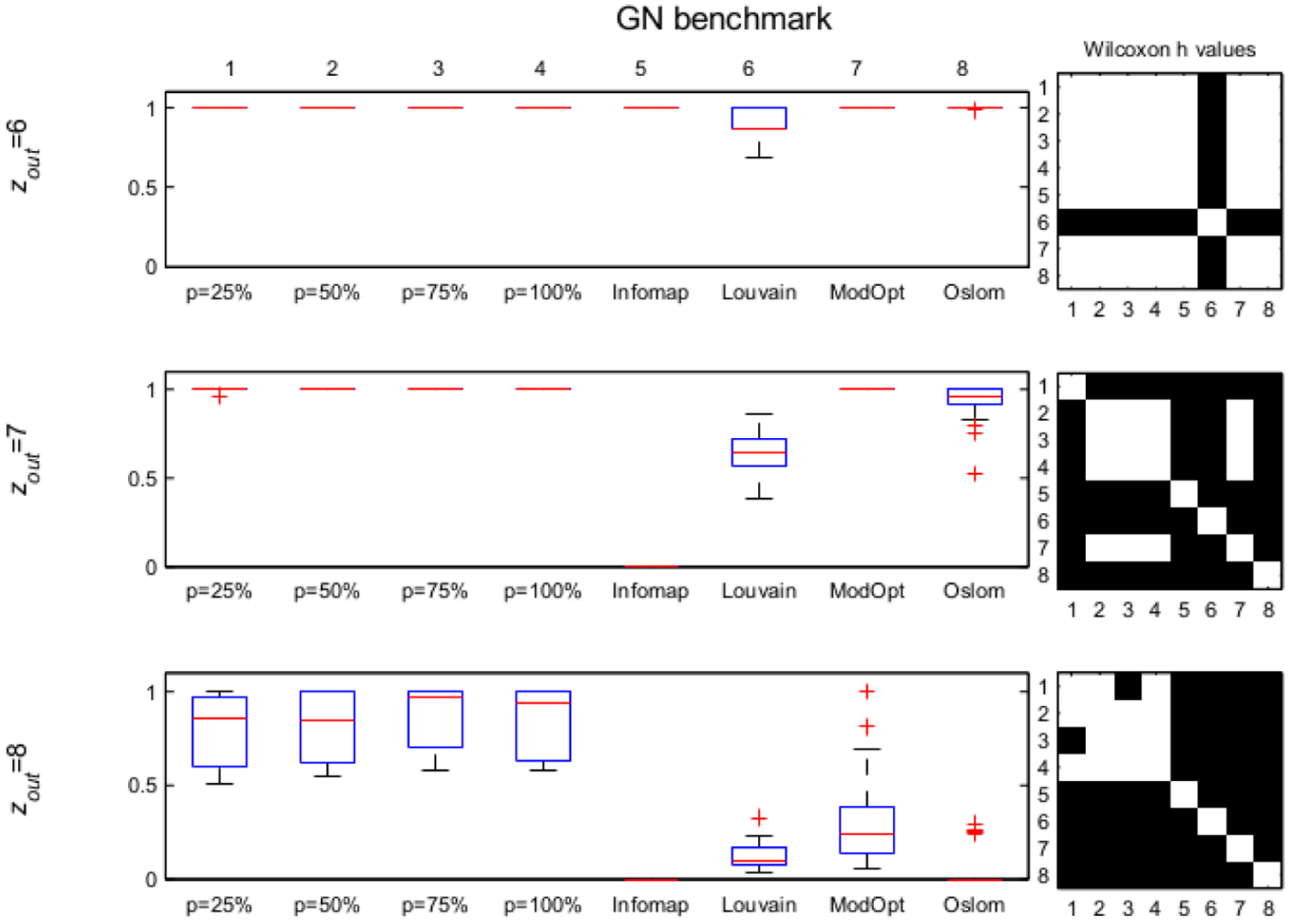
GN *z*_*out*_ = 6, 7, 8; boxplots of NMI values for all methods considered (left). On the right, the black-white matrix represents Wilcoxon *h* values for each pair of methods considered, numbered in the order they appear in the boxplot. A black square indicates a statistical difference between results. For *z*_*out*_ = 8 results obtained with all values of *p* are significantly better than all other methods considered.

Results obtained on networks with clear community structures are presented in the Supporting information. Thus, for the GN benchmarks with *z*_*out*_ ≤ 5 all methods compute the correct structure (S1 Fig). For *z*_*out*_ = 6 and 7 (Fig 6), *p*MNEO correctly identifies the structure for all values of *p*, with the exception of one network for *p* = 25% and *z*_*out*_ = 7. For *z*_*out*_ = 8, which is the most difficult set in the GN benchmark, all results obtained by *p*MNEO are significantly better than those obtained by the other methods. Regarding other results in literature obtained with an EO based method [46], we find that they report the fraction of nodes correctly classified as 1 for *z*_*out*_ values from 1 to 6 and decreasing to approx. 0.8 to *z*_*out*_ = 8.

**Fig 7:**
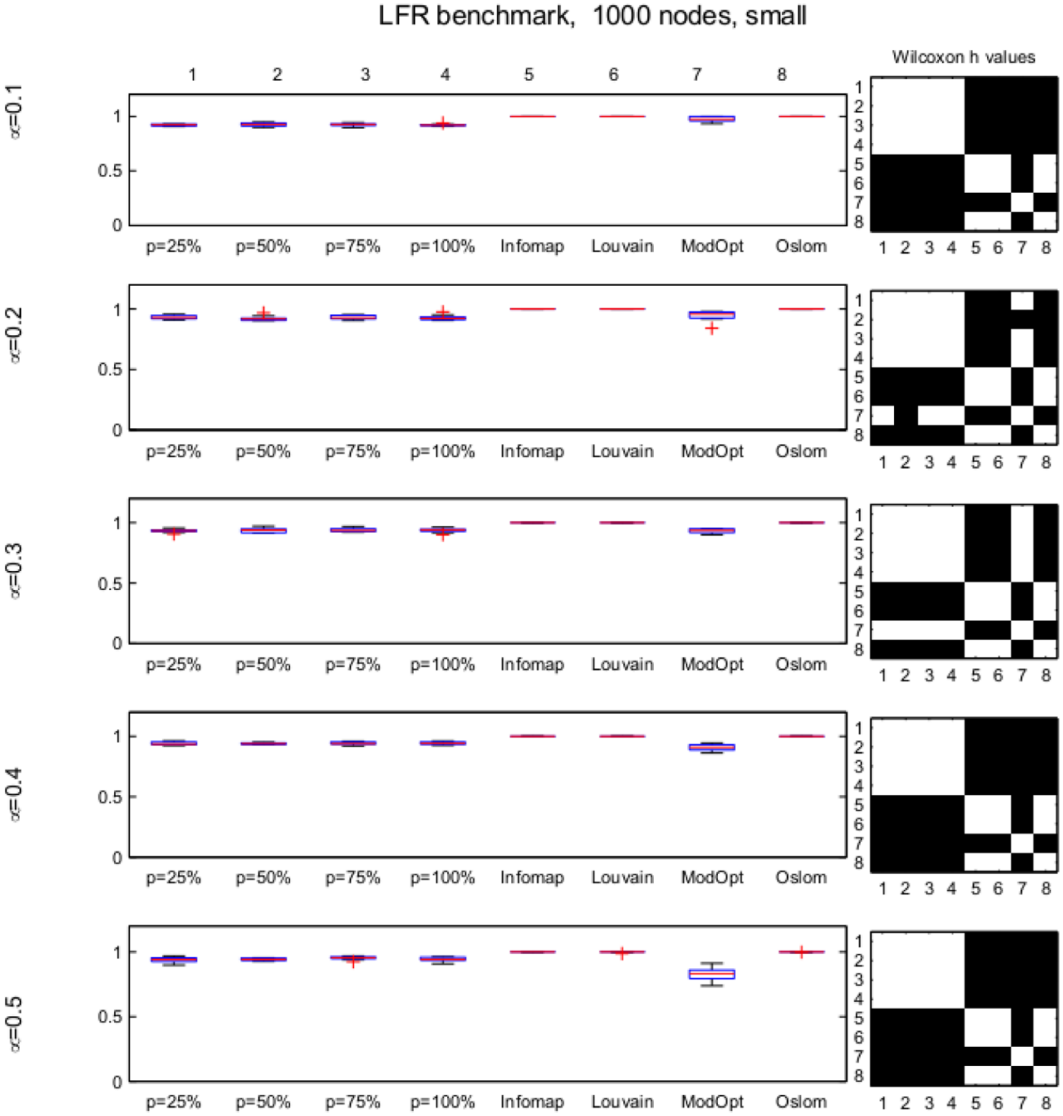
LFR small, 1000 nodes; boxplots of NMI values for all methods considered (left). On the right, the black-white matrix represents Wilcoxon *h* values for each pair of methods considered, numbered in the order they appear in the boxplot. A black square indicates a statistical difference between results.

For the LFR set with 128 nodes and *μ* ≤ 0.3, results are similar with those for the GN (S2 Fig). For the most difficult set, having *μ* = 0.5, *p*MNEO results are significantly better than those reported by Infomap and OSLOM. The results obtained for the LFR sets with 1000 nodes (Fig 7 and S3 Fig) show that all eight methods are competitive as they are all capable to identify the community structure with NMI values close to 1 (equal to 1 for Infomap).

**Fig 8:**
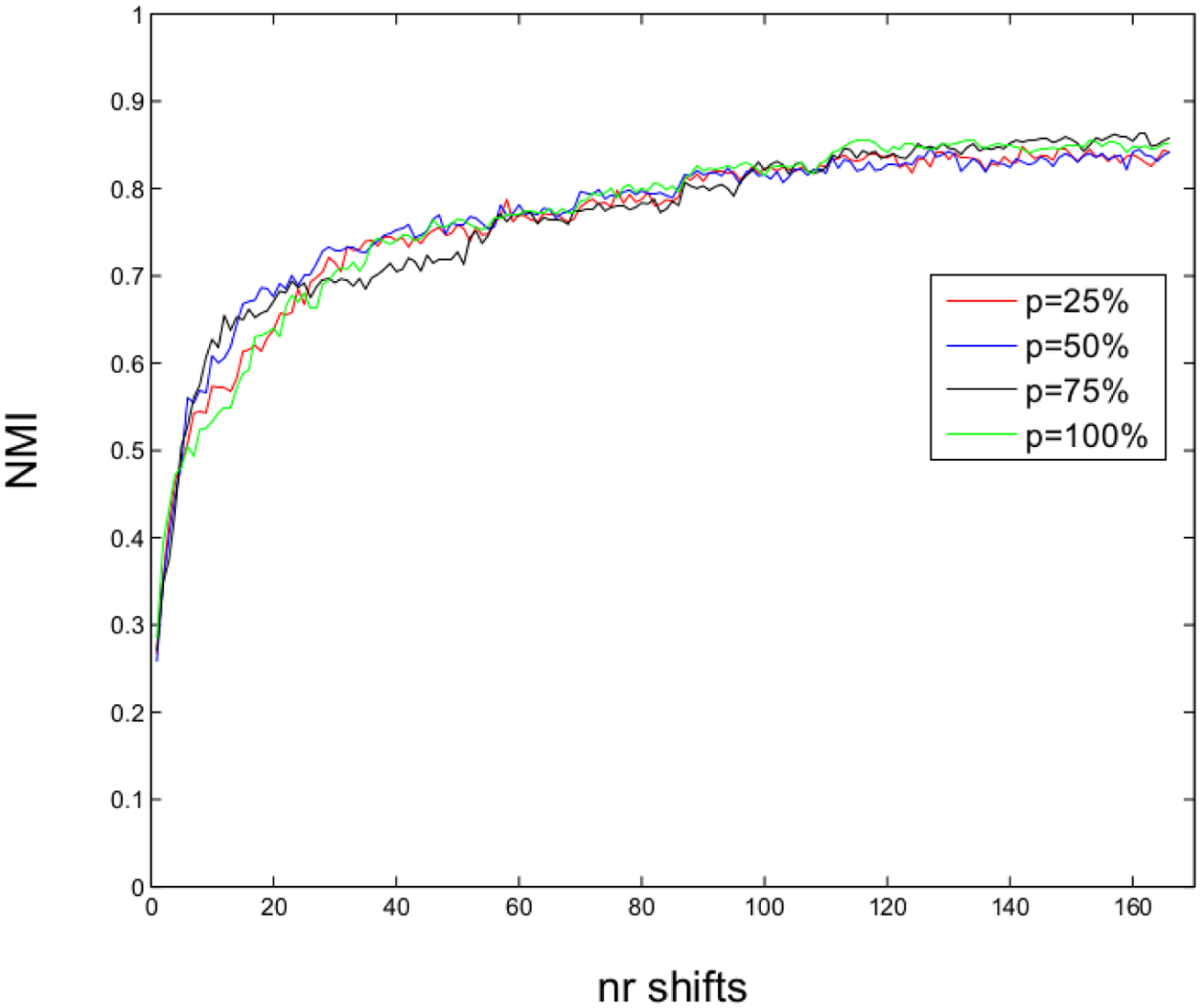
Evolution of the mean NMI values over time for the GN *z*_*out*_ = 8 set and different *p* values.

All results obtained on the synthetic networks show no significant difference among the values of the *p* parameter. Thus, we may conclude that, for networks presenting similar structures with the tested ones, the Nash ascendancy relation can be replaced with the reduced variant considering even only 25% of the nodes when comparing two strategy profiles. The evolution of NMI values over time for the GN *z*_*out*_ = 8 set, represented in Fig 8, shows that for this network set the evolution is similar, with overlapping trends, for all *p* values.

**Fig 9:**
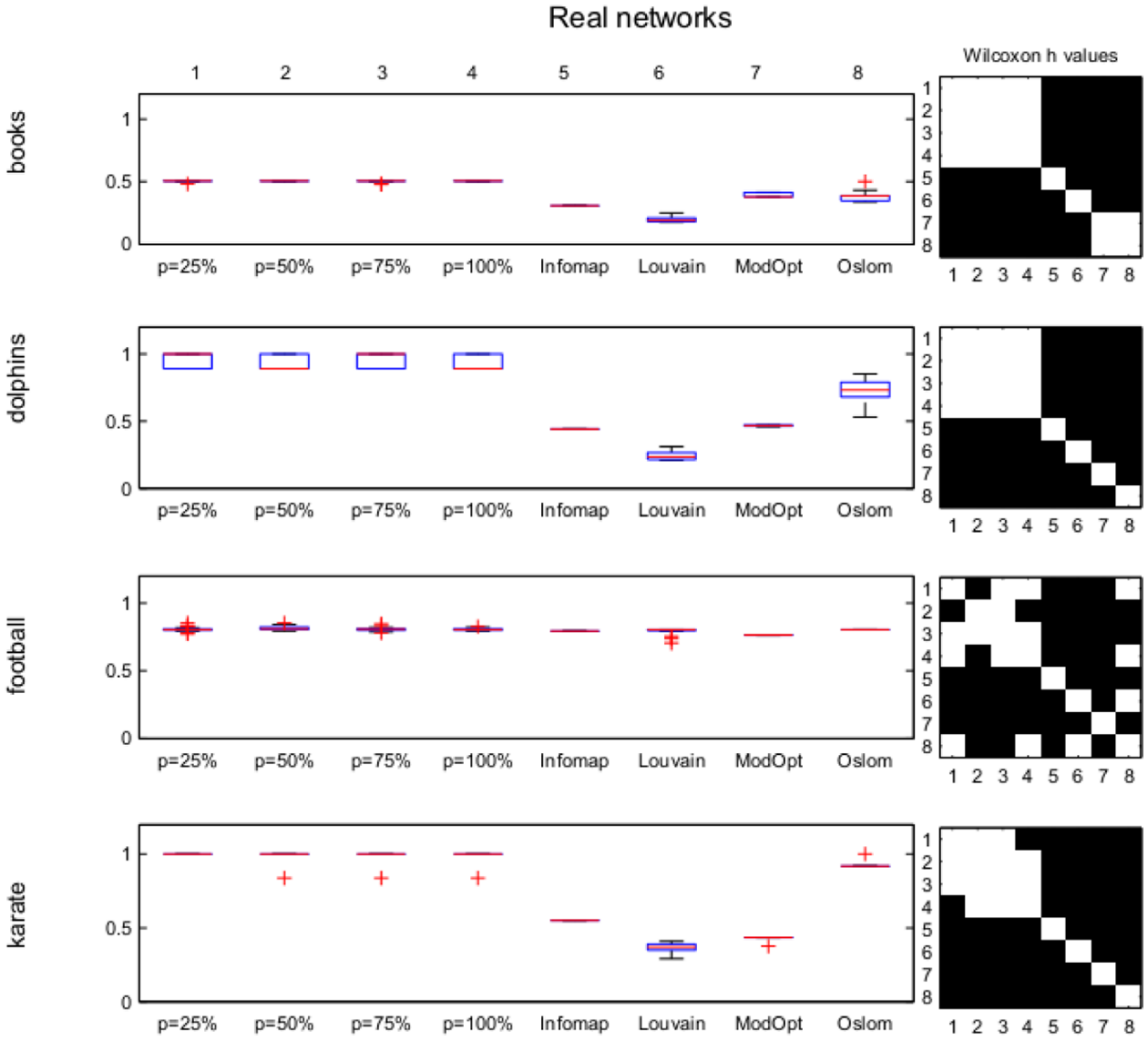
Real-world networks; boxplots of NMI values for all methods considered (left). On the right, the black-white matrix represents Wilcoxon *h* values for each pair of methods considered, numbered in the order they appear in the boxplot. A black square indicates a statistical difference between results.

Fig 9 presents the results obtained for the four real-world networks tested. They show that for all *p* settings, *p*MNEO results are the same or better than those obtained by the other methods. Again, there is no statistical difference among results obtained for different *p* values in most cases.

Most other methods based on game theory in literature do not present extensive numerical results to allow comparisons. In [28] experiments are performed on the GN and LFR 1000 S sets, using their method, SNS-CD, and also the Game algorithm in [21] and NashCoDe in [29]. On the GN set, SNS-CD reports a NMI equal to 1 for *z*_*out*_ ≤ 7 and decreases below 0.5 for *z*_*out*_ = 8. According to their results, Game and NashCoDE reported an NMI of 0 for all GN sets. In the case of LFR 1000 S, SNS-CD reports best results among game theoretic based approaches, with NMI values of approx. 0.95 for *μ* = 0.1, 0.2, and 0.85 for *μ* = 0.3, 0.4 and decreasing below 0.5 for *μ* = 0.5. *p*MNEO results are better than all these (Figs 6, 7 and S1 Fig) for all tested *p* values.

#### Is it Nash equilibrium?

While computing the Nash equilibrium is recognized to be challenging task, verifying that a strategy profile is a Nash equilibrium is a computationally expensive, yet trivial: we need to check for each player if unilateral deviations increase their payoff. When the strategy set is discrete, we can verify this by exhaustive search. To do so, for all results reported by *p*MNEO we counted the number of nodes (players) that would improve their payoffs by changing community. In order to compare results among networks with different sizes we divided this number to the network size. Results are presented in Tables 1 and 2.

**Table 1:** Fraction of nodes that can improve their payoffs by unilateral deviation in solutions reported by *p*MNEO for networks with known community structure. A zero value indicates that reported solutions are indeed Nash equilibria of game $\tilde{\Gamma }$. We report average values ± standard deviations and confidence limits of the mean.

*μ*		*p* = 0.25	*p* = 0.5	*p* = 0.75	*p* = 1
**GN**					
*z*_*out*_:1-8	mean ± stdev	0.00 ± 0.00	0.00 ± 0.00	0.00 ± 0.00	0.00 ± 0.00
	min	0.00	0.00	0.00	0.00
	CL mean	0.00 0.00	0.00 0.01	0.00 0.00	0.00 0.00
**LFR 128**					
0.1-0.5	mean ± stdev	0.00 ± 0.00	0.00 ± 0.00	0.00 ± 0.00	0.00 ± 0.00
	min	0.00	0.00	0.00	0.00
	CL mean	0.00 0.00	0.00 0.01	0.00 0.00	0.00 0.00
**LFR 1000 big**					
0.1	mean ± stdev	0.01 ± 0.02	0.00 ± 0.01	0.00 ± 0.01	0.01 ± 0.01
	min	0.00	0.00	0.00	0.00
	CL mean	0.01 0.02	0.00 0.01	0.00 0.01	0.00 0.01
0.2	mean ± stdev	0.00 ± 0.00	0.01 ± 0.02	0.00 ± 0.01	0.00 ± 0.01
	min	0.00	0.00	0.00	0.00
	CL mean	0.00 0.00	0.01 0.02	0.00 0.01	0.00 0.01
0.3	mean ± stdev	0.00 ± 0.01	0.00 ± 0.00	0.00 ± 0.00	0.00 ± 0.01
	min	0.00	0.00	0.00	0.00
	CL mean	0.00 0.01	0.00 0.00	0.00 0.00	0.00 0.01
0.4-0.5	mean ± stdev	0.00 ± 0.00	0.00 ± 0.00	0.00 ± 0.00	0.00 ± 0.00
	min	0.00	0.00	0.00	0.00
	CL mean	0.00 0.00	0.00 0.01	0.00 0.00	0.00 0.00
**LFR 1000 small**					
0.1	mean ± stdev	0.10 ± 0.05	0.11 ± 0.04	0.12 ± 0.04	0.17 ± 0.11
	min	0.05	0.05	0.07	0.04
	CL mean	0.07 0.14	0.08 0.14	0.09 0.15	0.09 0.24
0.2	mean ± stdev	0.14 ± 0.06	0.12 ± 0.06	0.11 ± 0.08	0.09 ± 0.05
	min	0.07	0.03	0.00	0.01
	CL mean	0.10 0.18	0.08 0.17	0.05 0.16	0.06 0.13
0.3	mean ± stdev	0.11 ± 0.07	0.10 ± 0.08	0.12 ± 0.09	0.08 ± 0.06
	min	0.03	0.01	0.05	0.00
	CL mean	0.06 0.16	0.04 0.15	0.05 0.18	0.04 0.12
0.4	mean ± stdev	0.07 ± 0.03	0.07 ± 0.04	0.05 ± 0.03	0.06 ± 0.03
	min	0.03	0.00	0.01	0.01
	CL mean	0.05 0.10	0.04 0.10	0.03 0.07	0.03 0.08
0.5	mean ± stdev	0.06 ± 0.04	0.04 ± 0.03	0.04 ± 0.03	0.05 ± 0.04
	min	0.02	0.01	0.01	0.01
	CL mean	0.03 0.08	0.02 0.06	0.02 0.07	0.02 0.08
**Real networks**					
books	mean ± stdev	0.00 ± 0.00	0.00 ± 0.00	0.00 ± 0.00	0.00 ± 0.00
	min	0.00	0.00	0.00	0.00
	CL mean	0.00 0.00	0.00 0.00	0.00 0.00	0.00 0.00
dolphins	mean ± stdev	0.00 ± 0.00	0.00 ± 0.00	0.00 ± 0.00	0.00 ± 0.00
	min	0.00	0.00	0.00	0.00
	CL mean	0.00 0.00	0.00 0.00	0.00 0.00	0.00 0.00
fotball	mean ± stdev	0.00 ± 0.00	0.00 ± 0.00	0.00 ± 0.00	0.00 ± 0.00
	min	0.00	0.00	0.00	0.00
	CL mean	0.00 0.00	0.00 0.00	0.00 0.00	0.00 0.00
karate	mean ± stdev	0.00 ± 0.00	0.00 ± 0.00	0.00 ± 0.00	0.00 ± 0.00
	min	0.00	0.00	0.00	0.00
	CL mean	0.00 0.00	0.00 0.00	0.00 0.00	0.00 0.00

**Table 2:** Fraction of nodes that can improve their payoffs by unilateral deviation in solutions reported by *p*MNEO for real world networks with unknown community structure. A zero value indicates that reported solutions are indeed Nash equilibria of game $\tilde{\Gamma }$. We report average values ± standard deviations and confidence limits of the mean also for modularity *Q*.

Network		*p* = 0.25	*p* = 0.5	*p* = 0.75	*p* = 1
riskmap	*Q*	0.62 ± 0.00	0.61 ± 0.00	0.61 ± 0.01	0.61 ± 0.00
	mean ± stdev	0.00 ± 0.00	0.00 ± 0.00	0.00 ± 0.00	0.00 ± 0.00
	min	0.00	0.00	0.00	0.00
	CL mean	0.00 0.00	0.00 0.00	0.00 0.00	0.00 0.00
jazz	*Q*	0.44 ± 0.00	0.44 ± 0.00	0.44 ± 0.00	0.44 ± 0.00
	mean ± stdev	0.00 ± 0.01	0.01 ± 0.01	0.01 ± 0.01	0.00 ± 0.01
	min	0.00	0.00	0.00	0.00
	CL mean	0.00 0.01	0.00 0.01	0.00 0.01	0.00 0.01
contiguous USA	*Q*	0.58 ± 0.00	0.58 ± 0.01	0.58 ± 0.01	0.58 ± 0.01
	mean ± stdev	0.01 ± 0.01	0.00 ± 0.01	0.01 ± 0.01	0.00 ± 0.01
	min	0.00	0.00	0.00	0.00
	CL mean	0.00 0.02	0.00 0.01	0.00 0.02	0.00 0.01

Results in Table 1 show that for all small networks the reported solutions are Nash equilibria for all *p* values. For the LFR sets with 1000 nodes, where the algorithm did not reach a NMI value of 1, there are nodes that can improve their payoffs by changing communities (more for LFR small). For the real world networks, we find that the solutions are Nash equilibria. Looking at Table 2 we find that even when the real community structure is not available, the provided solutions (with a high modularity value) are good approximations of Nash equilibria.

**Fig 10:**
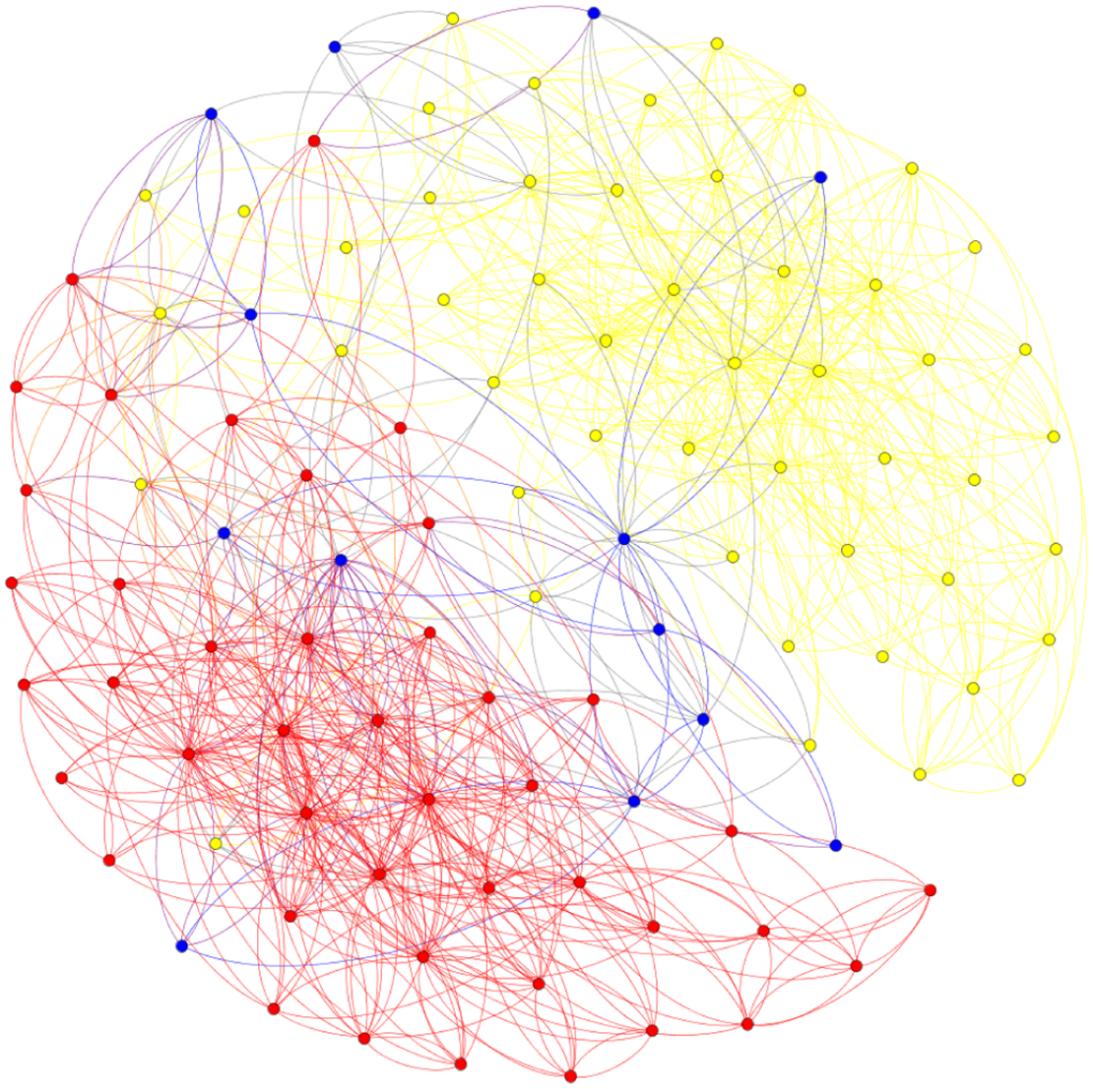
Political books dataset: The ‘real’ community structure is represented. We can see yellow nodes that have more colored links than yellow, indicating more links in the other two communities.

**Fig 11:**
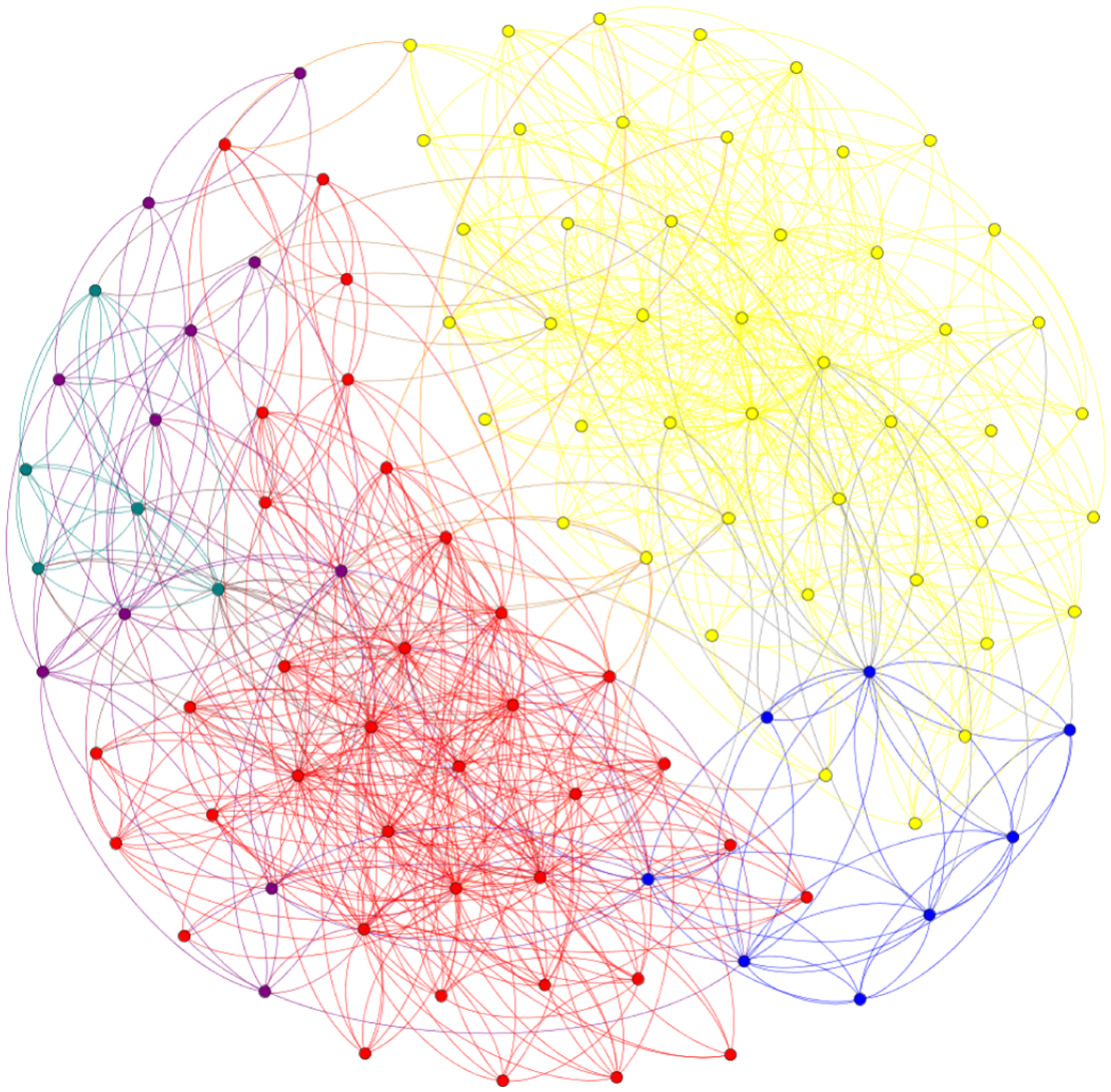
Political books dataset: A solution reported by *p*MNEO with NMI = 0.52 and verified to be a Nash equilibrium; some of the ‘difficult’ nodes are placed in a different community.

Furthermore, for real-world networks where the concept of ‘real community structure’ is debatable [47], these results that have the Nash equilibrium property of stability against unilateral deviations may actually provide a more realistic approach to this concept. For example, the *books* dataset is constructed from online data by considering books as nodes and edges are linking books that have been co-purchased by same buyers. The ‘real’ structure is considered to be set by the book’s political content. However, by studying the ‘real’ structure we can find nodes that have no link with other nodes in the community they are assigned to, which makes their ‘correct’ identification impossible (Figs 10 and 11). In this situation the equilibrium approach provides an alternative solution that may offer information about the real and not the expected structure of the network.

#### Efficiency

**Fig 12:**
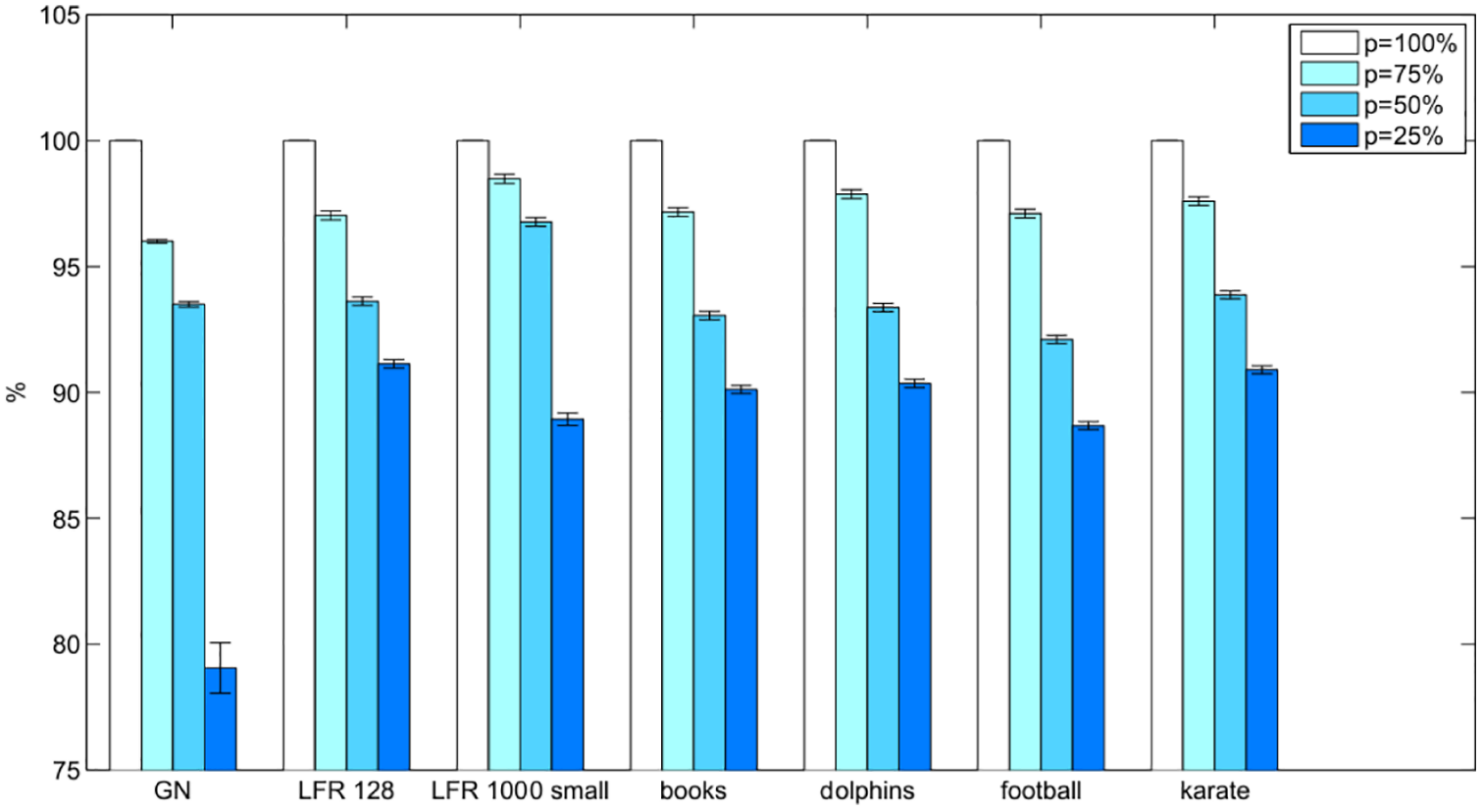
Differences in running times for the four tested *p* values as percentage of the running time for the *p* = 100% case for each network set.

One of the reasons the reduced Nash ascendancy relation was considered was to decrease the number of payoff function evaluations. Reducing this number up to only 25% should also reduce the running time of *p*MNEO. Fig 12 illustrates for each set the average duration necessary to run the experiments for different *p* values as percentage of the average duration for the *p* = 100% case. Results are similar for all the sets: reducing to 25% the percent of nodes that are used in the reduced ascendancy relation leads to an approximatively 10% decrease in running time. We may conclude that using the Nash ascendancy relation influences the running time less than other components of the extremal optimization algorithm.

**Fig 13:**
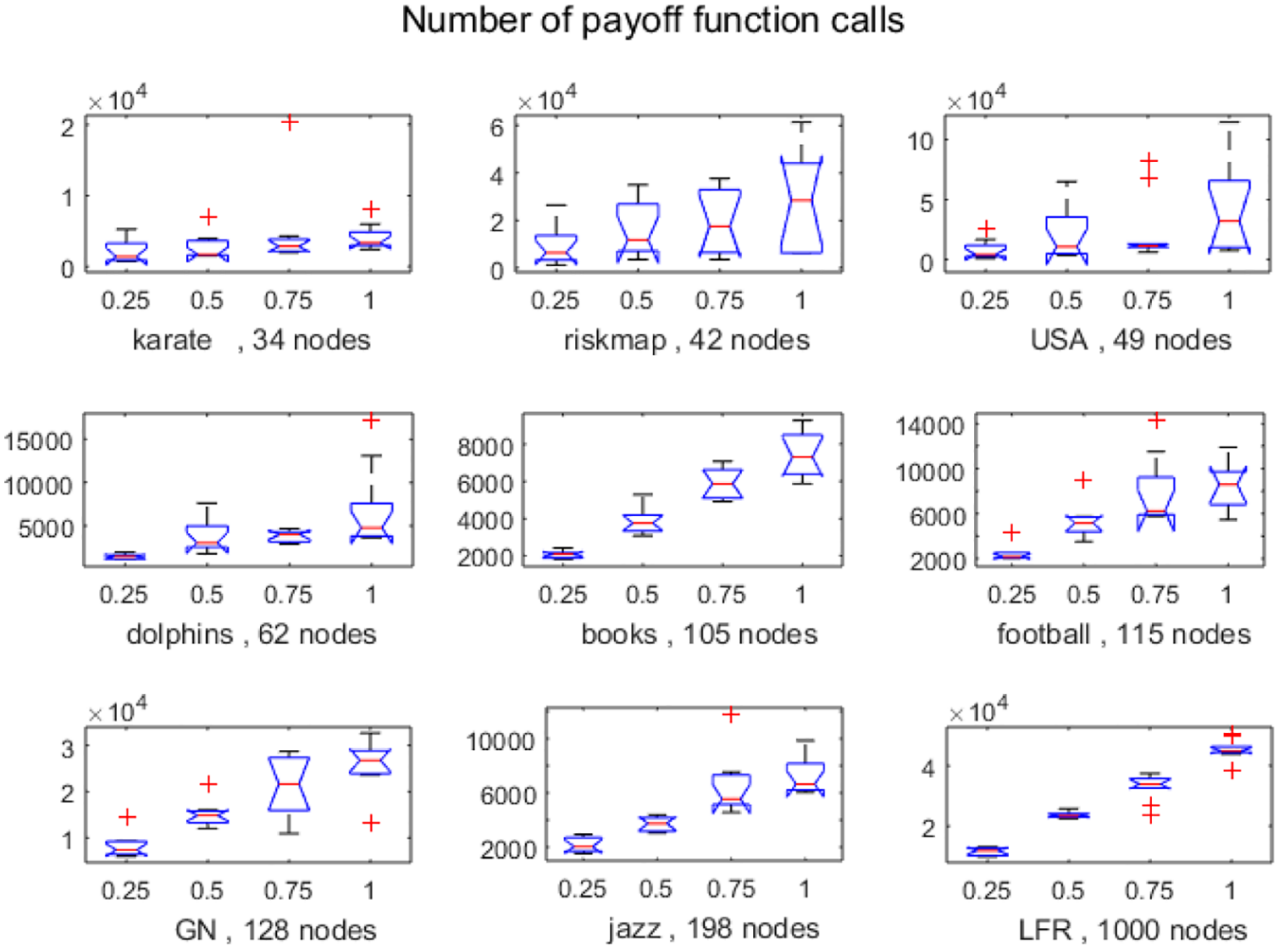
Number of payoff function calls in Alg. 2, 10 individuals. Notches indicate confidence limits of the mean.

In order to assess if there is indeed an economy in payoff function evaluations, we counted the number of payoff function calls that were actually performed from the *p*Nash ascendancy procedure (Alg. 2). Results are presented in Fig 13. The first thing to notice is that values reported for different *p* values on the same network do not have the same ratio between them as the *p* values themselves. The explanation for that is that the payoff function call in Alg. 2 is performed only if the selected player has different strategies in the two tested profiles (line 4). A significant difference would indicate the utility of considering the reduced generative relation for the community structure detection problem.

The Wilcoxon sum-rank test shows that the differences between *p* = 0.25 and *p* = 1 are significant for all networks (Table 3), while looking at NMI results reported for these values do not differ significantly (only in few cases). Table 3 also shows that most significant differences appear when comparing 0.25 with the others, and least differences between 0.75 and 1. These results show that even if the gain in running time -which also depends on the implementation—may not seem remarkable, there are benefits in using the reduced generative relation.

**Table 3:** Wilcoxon sum-rank results regarding differences in payoff function calls for the tested *p* values. An * indicates significant difference.

Network	0.25 → 0.5	0.25 → 0.75	0.25 → 1	0.5 → 0.75	0.5 → 1	0.75 → 1
karate	–	–	*	–	*	–
riskmap	–	–	*	–	–	–
USA	–	*	*	–	–	–
dolphins	*	*	*	–	*	*
books	*	*	*	*	*	*
football	*	*	*	*	*	–
GN	*	*	*	*	*	–
jazz	*	*	*	*	*	–
LFR 1000	*	*	*	*	*	*

Compared with the other methods, however, *p*MNEO is slower, like many other evolutionary techniques. A typical run for a network with 1000 nodes, population of 50 individuals and 6000 generations takes about one hour on a Intel(R) Core(TM) Quad CPU Q9400 @2.66GHz, while for Oslom and Infomap it takes minutes. However, the results obtained on difficult network show that *p*MNEO is a more refined method with potential to reveal structures that are not discoverable by other methods.

## Conclusion

We explore the possibility of using a reduced version of the Nash ascendancy relation within an extremal optimization method designed to identify the community structure of a network. The reduced ascendancy relation takes into account only a fraction of the network nodes when comparing two strategy profiles. We show that *p*–Nash non-ascended solutions are also Nash equilibria of the game. We address the practical concern that using the reduced relation may not yield results as good as the Nash ascendancy relation when maintaining the same number of iterations by using numerical experiments. Results show that there are very few significant differences in results between different values for the fraction of nodes and that the performance of *p*MNEO is significantly better than that of other state-of-the-art methods on the tested benchmarks.

Regarding the running time, the differences between different probability levels are low: an average decrease of 10% in running time when switching from *p* = 100% to *p* = 25%. This result indicates that the Nash ascendancy relation, while indeed computationally expensive, does not influence the running time of *p*MNEO as much as other components of the method. However, the reduced version does offer a less computational expensive alternative to the Nash ascendancy relation, that can be used to enhance other heuristics that attempt to solve the community structure detection problem using game theoretic approaches.

## Supporting information

S1 Fig GN *z*_*out*_ = {1, …, 5}; boxplots of NMI values for all methods considered (left). On the right, the black-white matrix represents Wilcoxon *h* values for each pair of methods considered, numbered in the order they appear in the boxplot. A black square indicates a statistical difference between results. As these networks have very well defined community structures, all methods are capable to identify the correct structure.
(EPS)

S2 Fig LFR small, 128 nodes; boxplots of NMI values for all methods considered (left). On the right, the black matrix represents Wilcoxon *h* values for each pair of methods considered, numbered in the order they appear in the boxplot. A black square indicates a statistical difference between results.
(EPS)

S3 Fig LFR big, 1000 nodes; boxplots of NMI values for all methods considered (left). On the right, the black-white matrix represents Wilcoxon *h* values for each pair of methods considered, numbered in the order they appear in the boxplot. A black square indicates a statistical difference between results.
(EPS)
